# Large Introns of 5 to 10 Kilo Base Pairs Can Be Spliced out in *Arabidopsis*

**DOI:** 10.3390/genes8080200

**Published:** 2017-08-11

**Authors:** Ning Chang, Qingqing Sun, Jinglei Hu, Chuanjing An, Hongbo Gao

**Affiliations:** College of Biological Sciences and Technology, Beijing Forestry University, Beijing 100083, China; ningjing890115@126.com (N.C.); sunqq@bjfu.edu.cn (Q.S.); hjlei@bjfu.edu.cn (J.H.); anchuanjing@163.com (C.A.)

**Keywords:** *Arabidopsis*, large intron, transposon, reverse transcription PCR

## Abstract

Most of the eukaryotic genes contain introns, which are removed from the pre-RNA during RNA processing. In contrast to the introns in animals, which are usually several kilo base pairs (kb), those in plants generally are very small, which are mostly from dozens of base pairs (bp) to a few hundred bp. According to annotation version 10.0 of the genome of *Arabidopsis thaliana*, there are 127,854 introns in the nuclear genes; 99.23% of them are less than 1 kb, and only 16 introns are annotated to be larger than 5 kb, which are extremely large introns (ELI) in *Arabidopsis*. To learn whether these introns are true introns or not and how large introns could be in *Arabidopsis*, RT-PCR analysis of genes containing these ELIs were carried out. The results indicated that some of these putative introns are indeed ELIs. These ELIs are mainly composed of transposons or transposable elements (TE), excepting one, whose counterparts are also very long in diverse plant species. Thus, this study confirms the existence of introns larger than 5 kb or even 10 kb in *Arabidopsis*.

## 1. Introduction

Introns are a common and important feature of eukaryotic genes. It is the nucleotide sequence within a gene that is spliced out during the maturation of RNA. The splicing of RNA is a part of RNA processing pathway after the transcription and before the translation. Through RNA splicing, introns are removed and the exons are ligated to form the mature RNA product. Introns are found in the genes of most organisms and many viruses and exist in a wide range of genes, including protein coding messenger RNA (mRNA), ribosomal RNA (rRNA), and transfer RNA (tRNA) [1]. Introns in multicellular eukaryotes are evenly distributed throughout genes, including protein coding region and 5’ and 3’ untranslated region (UTR) [2].

The lengths of introns vary widely among different organisms and different genes. Generally, the sizes of introns in animal genes are larger than those in plant genes. For example, human genes tend to have small exons separated by long introns with the mean and median size of 3356 and 1023 bp, respectively [3]. In contrast, analyses of genes in *Arabidopsis thaliana* and maize indicated that their introns are substantially shorter than those in humans. The mean and median lengths of introns are 168 and 100 bp in *Arabidopsis* [4]. In maize, the mean and median lengths of introns are 516 and 146 bp [5]. The average intron size in *Physcomitrella patens* is 311 bp [6]. Similar to plant introns, introns in *Schizosaccharomyces pombe* (93 bp on average), *Drosophila melanogaster* (564 bp on average), and *Caenorhabditis elegans* (467 bp on average) are also substantially shorter [7]. Variations of the lengths and structures of introns among different genomes suggest that extensive gain and loss of intron sequences is common during evolution.

In our studies, we found that some of the introns in *Arabidopsis* have a length of several kb, which are very large and rare for *Arabidopsis*. Therefore, we wanted to know how large the introns in *Arabidopsis* can be. In this study, we systematically analyzed the sizes of introns in the genome of *A. thaliana*. We found that although 99.23% of the introns are shorter than 1 kb, a very small part of the introns are annotated or predicted to be larger than 5 kb, and in very rare case, introns are annotated to be larger than 10 kb. Because these introns are extremely large in *Arabidopsis*, we wanted to investigate whether they are true introns or not. The existence of some of them was validated by reverse transcription PCR (RT-PCR) analysis and DNA sequencing in this study, and sequences of these introns were also analyzed to reveal their origin and evolution. The implications of the results are also discussed.

## 2. Materials and Methods 

### 2.1. Bioinformatics Analysis

Sequences of introns in *A. thaliana* and the related information were retrieved from the BLAST data sets at TAIR website (http://www.arabidopsis.org, version database 10). Statistical analysis of the length of the introns was carried out via Microsoft Excel. Gene structure analysis of *Arabidopsis* genes was done manually and assisted with the information from TAIR and SALK T-DNA express (http://signal.salk.edu/cgi-bin/tdnaexpress/). Primers from the exons before and after the annotated large intron were designed for RT-PCR analysis. Multiple protein sequence alignments were done with CLUSTAL W. To investigate the intron information in other species, BLAST against the genome sequences with *Arabidopsis* protein sequences was carried out with NCBI tBLASTN (http://blast.ncbi.nlm.nih.gov/Blast.cgi). The lengths of the introns were calculated manually based on the boundary of intron/exon defined by the RT-PCR product and compared with the genomic sequence.

### 2.2. Plant Materials and Growth Conditions

*A. thaliana* plants used in this study were Columbia-0 (Col-0) and Landsberg (L*er*) ecotypes. Seeds were sowed in soil or on Murashige and Skoog (MS) plates and then placed in the growth chamber under long-day conditions (16 h light/8 h of darkness) at 22 °C. Leaves of four-week-old plants grown in soil, floral tissues, and one-week-old seedlings grown on MS plates were used for RNA isolation.

### 2.3. Total RNA Isolation and RT-PCR Analysis

Total RNA was extracted by the RNeasy Plant Kit (Aidlab, Beijing, China). For RT-PCR analysis, total RNA was reverse-transcribed with RevertAid First Strand cDNA Synthesis Kit (Fermentas, Waltham, MA, USA). Complementary DNA (cDNA) of *AT3G60961*, *AT1G58602*, *AT3G05410*, and *AT5G13250* were amplified with primers indicated in Figure 2 and listed in the Table S1. PCR products were sequenced, and the sequences were aligned with the genome sequence and compared with the annotated sequence.

## 3. Results

### 3.1. Analysis of the Distribution of Intron Sizes in the Genome of Arabidopsis thaliana

**Figure 1:**
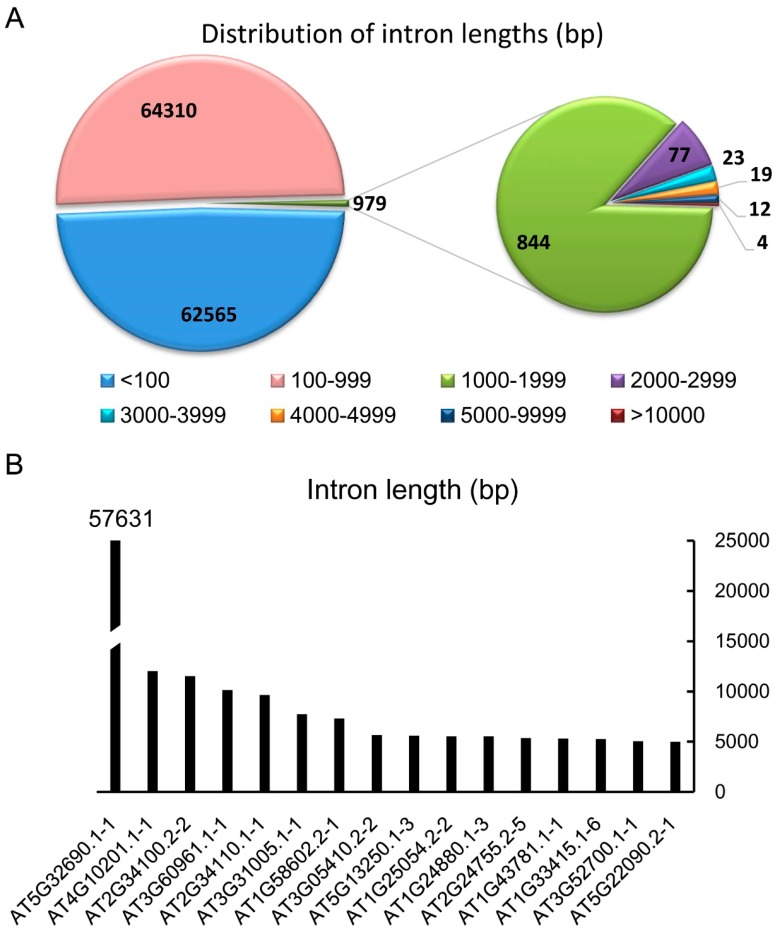
The distribution of intron lengths in *Arabidopsis thaliana* according to the annotation of TAIR10. (**A**) In the genome of *A. thaliana*, there are 127,854 introns of nuclear genes ranging from 8 bp to 57,631 bp. Among those, 62,565 introns are shorter than 100 bp, while 64,310 introns are from 100 to 999 bp. There are 844, 77, 23, 19, 12, and 4 introns in the range of 1000–1999 bp, 2000–2999 bp, 3000–3999 bp, 4000–4999 bp, 5000–9999 bp, and >10,000 bp, respectively. (**B**) A list of introns larger than 5000 bp.

According the annotation version TAIR 10 of the *Arabidopsis* genome, there are 127,854 introns in the nuclear genome. After analyzing the lengths of all the introns, we found that a large part (48.93%) of the introns is below 100 bp, and 50.30% of the introns are between 100 and 999 bp (Figure 1A). These introns constitute 99.23% of the introns in the genome of *Arabidopsis*. There are 979 introns larger than 1 kb, which is only 0.77% (a very small part) of the total introns. Introns larger than 3 kb are very rare, with only 58 in the nuclear genome. There are 16 introns annotated to be larger than 5 kb, which we labeled as extremely large introns (ELI) in *Arabidopsis* in this study (Figure 1B). Among those 16 ELIs, eight are located in protein-coding genes, four are in pseudogenes, two are in potential natural antisense genes, and two are obsolete or no longer exist (Table 1 and Table S2). AT1G24880.1-3 and AT1G25054.1-3 are obsolete and not investigated in this study. These annotated ELIs often contain transposons or transposable elements (TEs). Some of them also contain one or more genes. Rarely, some of the intron sequences are just non-repetitive sequences.

### 3.2. Validation and Sequence Analysis of Extremely Large Introns in Arabidopsis

**Figure 2:**
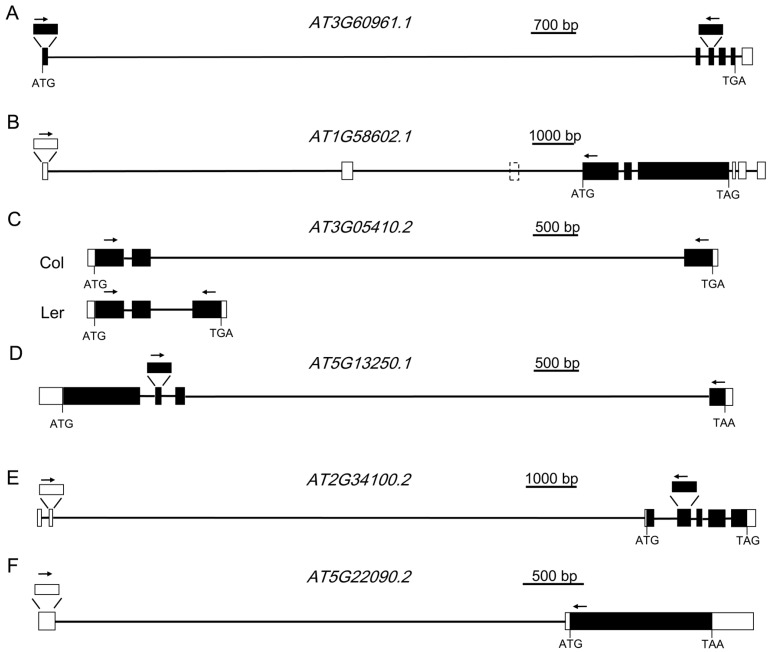
Gene structures of *AT3G60961.1*, *AT1G58602.1*, *AT3G05410.2*, *AT5G13250.1*, *AT2G34100.2*, and *AT5G22090.2*. (**A**) The structure of *AT3G60961.1*. (**B**) The structure of *AT1G58602.1*. (**C**) The structure of *AT3G05410.2* in Col and L*er* ecotype. (**D**) The structure of *AT5G13250.1*. (**E**) The structure of *AT2G34100.2*. (**F**) The structure of *AT5G22090.2*. White boxes represent the 5’ and 3’ untranslated region (UTR), black boxes represent the coding sequence, and black lines represent the intron. Arrows indicate the positions of forward and reverse primers for RT-PCR analysis.

**Figure 3:**
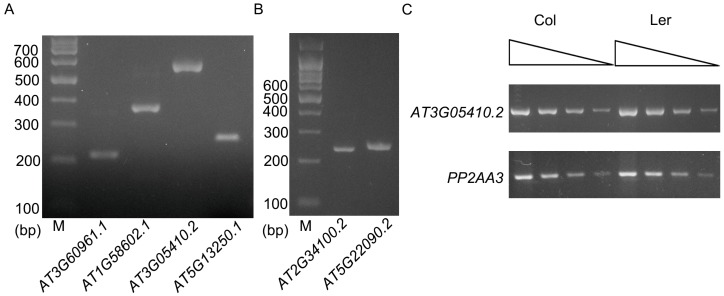
RT-PCR analysis of the genes shown in Figure 2. (**A**) RT-PCR analysis of *AT3G60961.1*, *AT1G58602.1*, *AT3G05410.2*, and *AT5G13250.1* with RNA extracted from leaves. Positions of primers are shown in Figure 2. (**B**) RT-PCR analysis of *AT2G34100.2* and *AT5G22090.2* with RNA extracted from floral tissues. Positions of primers are shown in Figure 2. (**C**) Semi-quantitative RT-PCR analysis of *AT3G05410.2* in Col and L*er* ecotype. Black triangles above indicate that the quantity of cDNA was serially diluted three times with a dilution factor of four (from left to right). *PP2AA3* gene (*AT1G13320*) was used as a control.

To learn whether these ELIs are true introns or not, eight ELIs, the ones located in the protein-coding genes, were chosen for validation by RT-PCR and sequencing analysis (Table 1, Figure 2 and Figure 3). RT-PCR analysis of the RNA extracted from four-week old leaves (Figure 2A) validated the ELIs in *AT3G60961.1 AT1G58602.1 AT3G05410.2 AT5G13250.1.* A further RT-PCR analysis of the RNA extracted from floral tissues (Figure 2B) validated the ELIs in *AT2G34100.2* and *AT5G22090.2*. The annotated ELI in *AT3G52700.1* could not be validated (Figure S1). The RT-PCR products of *AT2G34110.1* were sequenced and found to be an unrelated sequence. Overall, six of them were validated, two of them were not, and a new ELI was discovered (Figure 2).

*AT3G60961.1* encodes a protein of the P-loop containing nucleoside triphosphate hydrolases superfamily. The first intron of this gene, AT3G60961.1-1, was annotated to be 10,233 bp. A pair of primers (Figure 2A) located at the two exons before and after this intron was designed to validate the existence of this intron by RT-PCR analysis. As shown in Figure 3A, PCR products with an expected size were amplified. Further sequencing analysis indicated that the transcript is almost the same as the one annotated, except the 5’ splicing site of the intron is 13 bp upstream. This intron was also annotated as *AT3G60965*, which is a copia-like retrotransposon and constitutes 96.6% of the intron. There are also transposable elements before and after this gene.

*AT1G58602.1* encodes a disease resistance protein with LRR and NB-ARC domains. The 5’-UTR of this gene was annotated to have three introns, with the first intron found to be 7384 bp. A pair of primers located at the first and the fourth annotated exon (Figure 2B) was designed to validate the existence of this intron by RT-PCR analysis. DNA sequencing results validated the first intron. Surprisingly, the third exon was missing in the transcript, suggesting that the second intron, the second exon, and the third intron are all spliced out. Therefore, this intron, 6070 bp in size, is a newly discovered extremely large intron in *Arabidopsis*. The first intron contains *AT1G58561*, a copia-like retrotransposon, and several other TEs. The second intron reported here also contains several TEs. The genomic regions before or after this gene also contain multiple TEs.

*AT3G05410* encodes a Photosystem II reaction center protein, OEC23 (or PsbP). It has multiple alternative splice variants. The second intron of *AT3G05410.2* is 5748 bp long, and was validated by our RT-PCR analysis. This intron is mainly composed of *AT3TE06550*, which belongs to non-LTR retrotransposon family (LINE). The protein AT3G05410 and its closest homolog in *Arabidopsis*, AT3G55330, have an identity of 25% and an *E* value of 5 × 10^−10^ when a BLAST search was done at TAIR. However, it was shown to have an identity of 86% and an *E* value of 2 × 10^−172^ with its homolog in *Raphanus sativus* in a BLAST search at the National Center for Biotechnology Information (NCBI). This suggests *AT3G05410* is a single copy gene in the genome of *Arabidopsis* and it is an essential gene for photosynthesis. The exon after this intron is not included in *AT3G05410.1*. However, the coding sequence of this exon is well conserved in different plant species (Figure S2), suggesting this large intron should be spliced out for the normal function of the gene. This intron in the L*er* ecotype is only 456 bp, which has a left border and a right border similar to that of Col ecotype. Moreover, this intron in *Arabidopsis lyrata* and many other plant species is also hundreds of bp (Table 2). These data suggest that recently after the divergence of Col and L*er* ecotypes, a transposon was inserted in this intron. A semi-quantitative RT-PCR analysis indicated that the presence of this large intron has no large effect on the mRNA level of this gene (Figure 3C).

*AT5G13250.1* encodes a RING finger protein. The third intron of this gene, AT5G13250.1-3, was annotated to be 5670 bp. Like *AT3G05410.2*, the protein sequence encoded by the exon after this intron is well-conserved in different plants (Figure S3). RT-PCR was carried out to validate this intron. Sequencing result indicated that this intron is 3 bp into the predicted following exon. This intron was also annotated to have several short transposable elements. Unlike the large introns analyzed above, the overall sequence of this intron is not a transposon or repetitive sequence, which are only 530 bp and constitute 9.3% of the intron. Analysis of the homologs of this gene in various plant species, including rice, suggests that they all have a large intron of several kb or even more than 10 kb at the corresponding location (Table 3). During evolution, this intron remained very large.

*AT2G34100* encodes a protein with homologs only found in some species of the Brassica family. *AT2G34100.1* and *AT2G34100.2* encode the same protein sequence. *AT2G34100.2* has a longer 5’-UTR, whose coding sequence is far upstream. The second intron of *AT2G34100.2* is 11,602 bp, and was validated by our RT-PCR analysis (Figure 2E). According to the annotation, in this intron, there are 13 genes or TEs. Ten of them are in the reverse direction of *AT2G34100.* The longest one among those, *AT2TE63800*, is 4541 bp.

*AT5G22090* encodes a protein with a Fantastic Four (FAF) domain. FAF belongs toa family of proteins that regulate the size of the plant shoot meristem by modulating the CLV3-WUS feedback loop. Similar to *At2G34100*, *AT5G22090* was annotated to have two splice variants that encode the same protein sequences. *AT5G22090.2* has a large intron of 5081 bp (Figure 2F). In this intron, there are three transposable elements, which are all in the reverse direction of the gene.

## 4. Discussion

In this study, we show clear evidence that some introns in *Arabidopsis* could be much larger than usual. Introns in *Arabidopsis* are mostly 50 to 300 bp, which account for 87.04% of the total introns. Introns larger than 1 kb account only for 0.77% of all the introns, and there are only 16 annotated introns larger than 5 kb in the whole genome. We chose eight of them for validation, and found that six of them are true introns. We also discovered an additional large intron that is 6070 bp length. These data suggest that the actual genetic structures in *Arabidopsis* could be more complicated than what is thought, and some genes with extremely large introns could be misannotated or overlooked. A misannotation of the large intron between the 5’ UTR sequences could give the wrong position of the promoter of the gene, while a misannotation of the large intron between the coding sequences could cause the annotation to miss a part of the gene.

Six out of the seven ELIs reported in this study are mainly transposons, multiple transposons (i.e., transposons in transposons), or full of TEs. It is well known that the genome of *Arabidopsis* is very compact, and only a very small part of its genome is composed of transposons. The genome of many other species, including other plants, have a much higher presence of transposons [3,5,8]. Transposon insertions and DNA sequence translocations into introns could increase the complexity of the genome and the difficulty of genome annotation, especially for higher eukaryotic species other than *Arabidopsis*.

Introns in the genome of *Arabidopsis* and other plants are usually small (shorter than a few hundred base pairs) [4,5,6,9]. In extreme cases, an intron could be only 1 bp [10]. Our results suggest that transposon insertions or DNA translocations could increase the size of introns to several kb or even larger. These sequences could provide the materials for genome evolution, increase the complexity of gene structures, and finally be functional, as large introns could be important for the regulation of gene expression and function [11,12,13]. In the case of AT5G13250.1-3, all the introns at this position in flowering plants are very large, suggesting they may have functional elements. On the other hand, small introns seem to be common in most of the genes. During evolution, larger introns might have become smaller through DNA deletion during DNA replication if they do not have functional elements, since introns with a smaller size were more favored by the genome.

Our results show that the insertion of transposons in the introns of some genes might have little or no effect on the splicing of the pre-mRNA. Mutants caused by transfer-DNA (T-DNA) and transposon insertions are widely used in the study of *Arabidopsis* and other plant species [14,15,16,17]. These insertions are usually only several kb, which are comparable to or even smaller than the introns reported in this study. Often, we can see T-DNA and transposons inserted in introns of genes in the insertional mutants of *Arabidopsis* (http://signal.salk.edu/cgi-bin/tdnaexpress/). These mutants were once seen as null alleles, although it is conceivable that some of the insertions in the introns could abolish the splicing, our study also suggests that some of the introns with insertions of several kb length could be spliced out with little or no problem.

## Figures and Tables

**Table 1:** An analysis of the introns larger than 5 kb in protein coding genes in *A. thaliana*.

Gene	Annotated Intron	Length (bp)	Validation Results	Description of Gene	Description of Intron	Total Size of TEs in the Intron
*AT2G34100.2*	AT2G34100.2-2	11,602	Validated; 11,602 bp	Nonsense-mediated mRNA decay-like protein	This intron contains 13 genes or TEs: *AT2TE63770, AT2G34110, AT2TE63775, AT2TE63780, AT2G34120, AT2TE63785, AT2G34123, AT2TE63790, AT2TE63795, AT2TE63800, AT2G34130, AT2TE63805, AT2G34135*, with different sizes.	6325 bp; (54.52%)
*AT3G60961.1*	AT3G60961.1-1	10,234	Validated; 10,247 bp	P-loop containing nucleoside triphosphate hydrolases superfamily protein	This intron was also annotated as AT3G60965, a copia-like retrotransposon.	9903 bp (96.64%)
*AT2G34110.1*	AT2G34110.1-1	9724	Not validated	hypothetical protein	NA	
*AT1G58602.1*	AT1G58602.1-1	7384	Validated; 7385 bp	LRR and NB-ARC domains-containing disease resistance protein	This intron contains 4 TEs: *AT1TE71765, AT1TE71770, AT1TE71775* and *AT1TE71780*.	6164 bp (83.47%)
*AT1G58602.1*	AT1G58602.1-2 and AT1G58602.1-3	6070	Validated; 6070 bp	LRR and NB-ARC domains-containing disease resistance protein	These two annotated introns and the exon between them were found to be an intron of 6070 bp. It contains 10 TEs: *AT1TE71785, AT1TE71790, AT1TE71795, AT1TE71800, AT1TE71805, AT1TE71810, AT1TE71815, AT1TE71820, AT1TE71825* and *AT1TE71830*.	5192 bp (85.54%)
*AT3G05410.2*	AT3G05410.2-2	5748	Validated; 5748 bp	Photosystem II reaction center OEC23 protein	A major part of the intron is a transposon, *AT3TE06550*.	5261 bp (91.53%)
*AT5G13250.1*	AT5G13250.1-3	5670	Validated; 5673 bp	RING finger protein	This intron contains *AT5TE15340, AT5TE15345, AT5TE15350* and *AT5G02075*, which constitute only a small part of the intron.	530 bp (9.34%)
*AT3G52700.1*	AT3G52700.1-1	5134	Not validated	Hypothetical protein	NA	
*AT5G22090.2*	AT5G22090.2-1	5082	Validated; 5082 bp	FAF-like protein	This intron contains 3 TEs: *AT5TE26450, AT5TE26455, AT5TE26460*	1985 bp (39.06%)

TE: transposable element; mRNA: messenger RNA; LRR: leucine-rich repeat; NB: Nucleotide Binding; ARC: Apaf-1, certain R gene products, and CED-4; OEC: Oxygen-Evolving Complex; RING: Really Interesting New Gene; FAF: Fantastic Four; NA: not applicable.

**Table 2:** Lengths of intron AT3G05410.2-2 and its counterparts in other plants.

Organism	Length (bp)
*Arabidopsis thaliana* (Col-0)	5748
*Arabidopsis thaliana* (L*er*)	456
*Arabidopsis lyrata*	461
*Capsella rubella*	391
*Citrus sinensis*	77
*Populus euphratica*	83
*Vitis vinifera*	18
*Oryza sativa*	254
*Populus trichocarpa*	130
*Sesamum indicum*	2903

**Table 3:** Lengths of intron AT5G13250.1-3 and its counterparts in other plants.

Organism	Length (bp)
*Arabidopsis thaliana* (Col-0)	5670
*Arabidopsis lyrata*	8873
*Populus euphratica*	6986
*Citrus sinensis*	5302
*Vitis vinifera*	6587
*Oryza sativa* (Chromosome 4)	9945
*Oryza sativa* (Chromosome 12)	16,721
*Populus trichocarpa* (linkage group 1)	6684
*Populus trichocarpa* (linkage group 3)	6244
*Sesamum indicum*	5931

## Supplementary Materials

Supplementary files can be found at www.mdpi.com/2073-4425/8/8/200/s1. Figure S1: Gene structure and RT-PCR analysis of *AT3G52700.1*, Figure S2: Protein sequence alignment of AT3G05410.2 and its homologs in other species, Figure S3: Protein sequence alignment of AT5G13250.1 and its homologs in other species, Table S1: Sequences of the primers used in this study, Table S2: Annotated introns larger than 5 kb in genes that are not protein coding in *Arabidopsis thaliana*.
