# Supplementary material for: Large Introns of 5 to 10 Kilo Base Pairs Can Be Spliced out in Arabidopsis
Source: Genes (Basel). 2017 Aug 11;8(8):200. doi: 10.3390/genes8080200 (PMC5575664; doi:10.3390/genes8080200)
Supplement: Supplementary file 1 [file genes-08-00200-s001.docx]

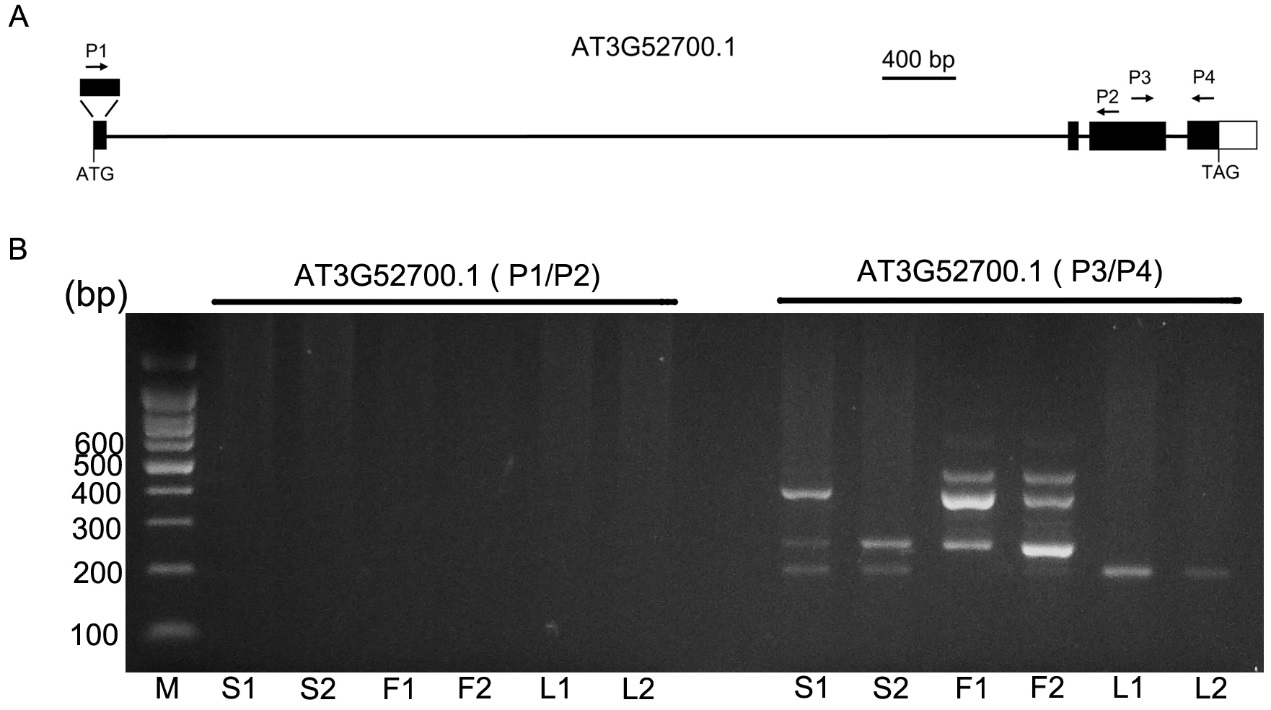


**Figure S1.** Gene structure and RT-PCR analysis of *AT3G52700.1*. **A** The annotated structure of *AT3G52700.1*. White boxes represent 3’ UTR, black boxes represent the coding sequence and black lines represent the intron. Arrows indicate the positions of the primers P1, P2, P3 and P4 for RT-PCR analysis. **B** RT-PCR analysis of *AT3G52700.1*. M represents maker. S, F and L represent seedlings, flowers and leaves, respectively. All the analysis was done with biological duplicates.


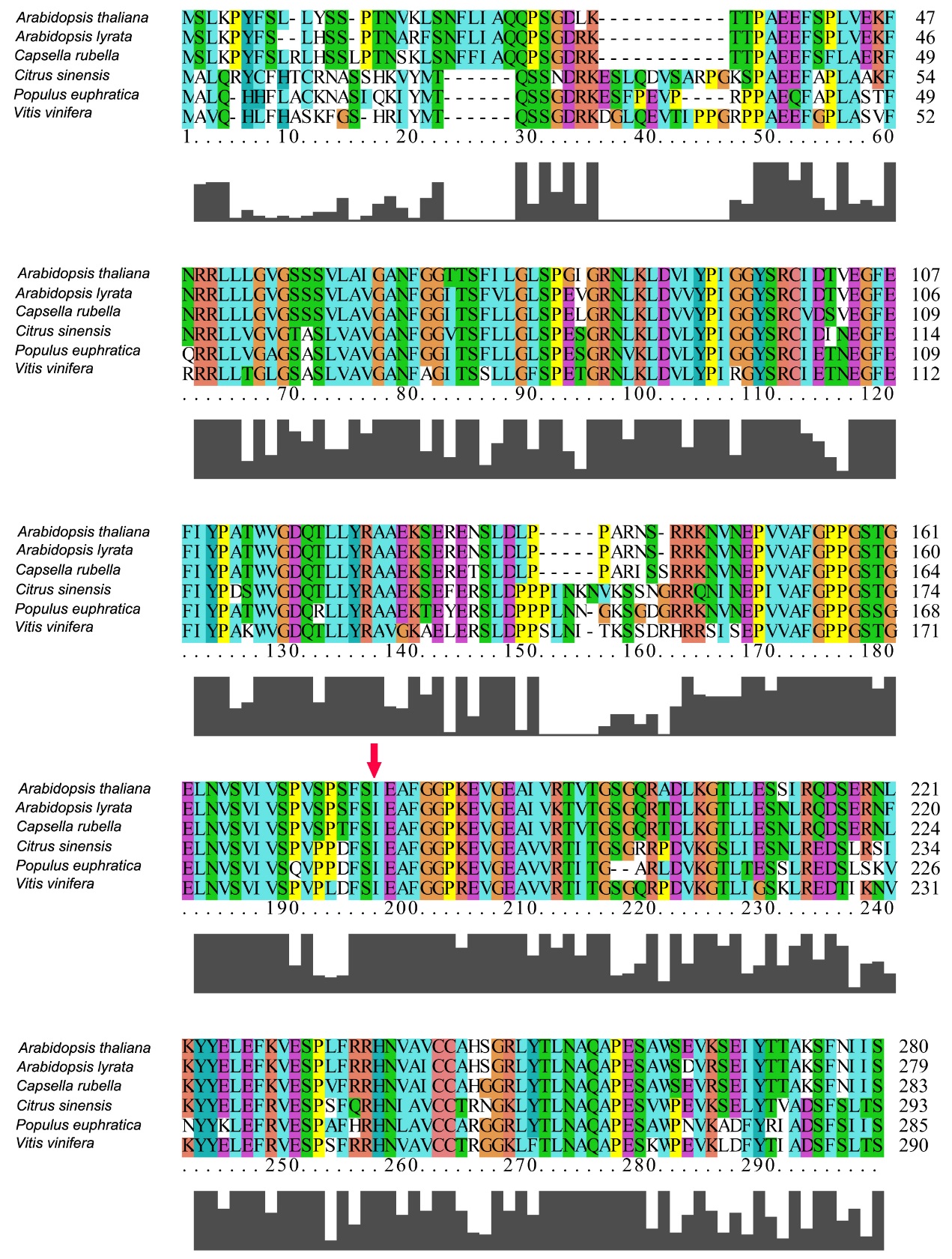


**Figure S2.** Protein sequence alignment of *AT3G05410.2* and its homologs in other species. The red arrow indicates the position of intron splicing site.


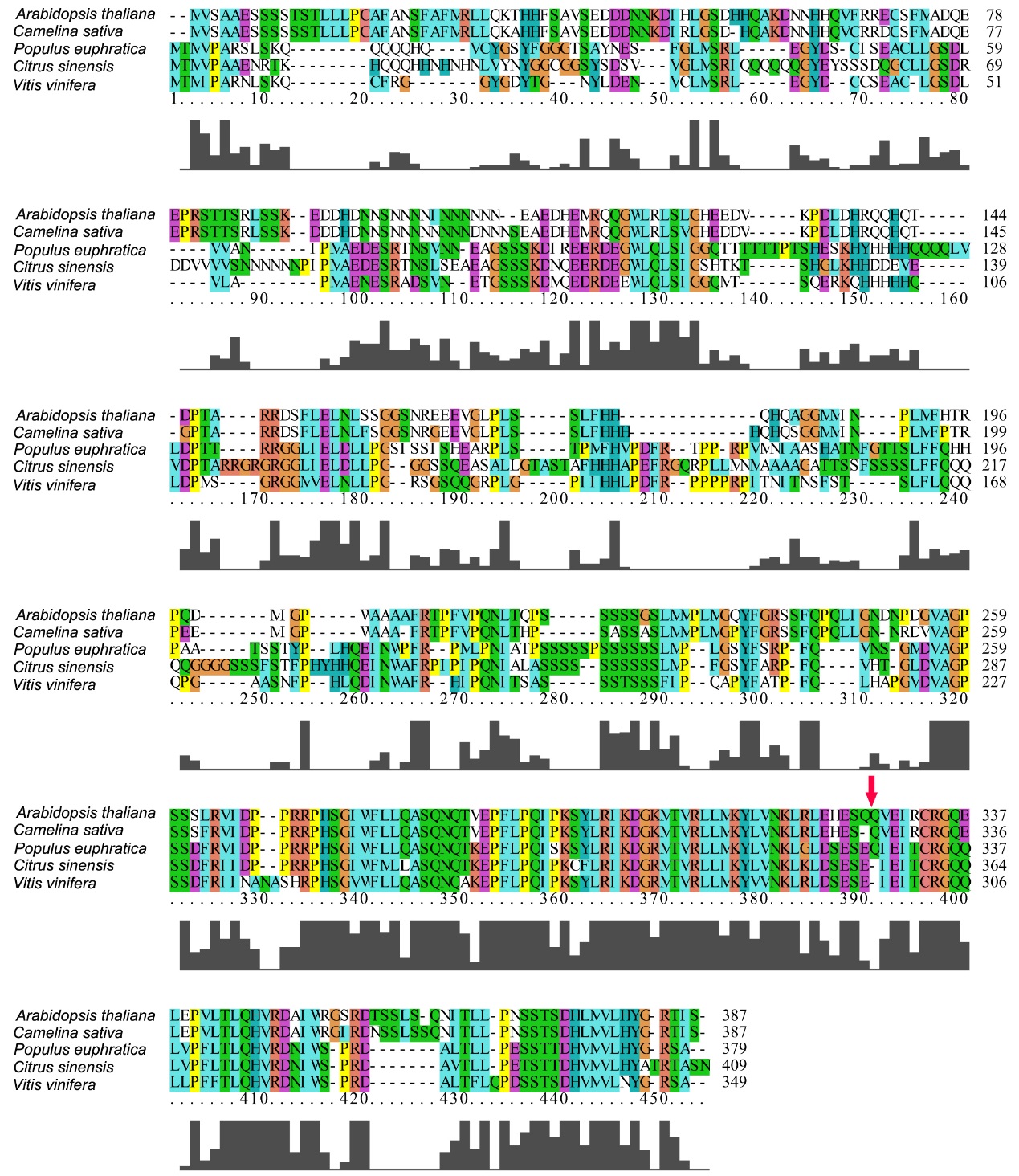


**Figure S3.** Protein sequence alignment of *AT5G13250.1* and its homologs in other species. The red arrow indicates the position of intron splicing site.

**Table S1.** Sequences of the primers used in this study.

| **Gene Name** | **Primer Sequences** |
| --- | --- |
| *AT2G34100.2* | Forward: 5’-GAT CTA TTG ATG GGA GAC TAC G-3’  Reverse: 5’-CCA TTA TCT GGC TGT CCG-3’ |
| *AT3G60961.1* | Forward: 5’-GAT TGC AGA GGG TAG AAT AGT G-3’  Reverse: 5’-GGT TCC TGC TCA TAA TGC G-3’ |
| *AT2G34110.1* | Forward: 5’-CGA AGC TAT ATC AGA AGA AAG TG-3’  Reverse: 5’-CTC CGT CGT CAT CAT CGC-3’ |
| *AT1G58602.1* | Forward: 5’-CCT CTT CTT CCT AAA CGT CG-3’  Reverse: 5’-CAG CTA CTT GAT CTT CGA CTC-3’ |
| *AT3G05410.2* | Forward: 5’-GGC TTC TTC TGG GTG TAG-3’  Reverse: 5’-GCT ACA TTA TGC CGC CGG-3’ |
| *AT5G13250.1* | Forward: 5’-CCA TTC TTG CCT CAG ATT CC-3’  Reverse: 5’-GAG GTA GAT GAG TTT GGA AGC-3’ |
| *AT3G52700.1* | P1: 5’-AAA TAG ATC GTT CGT ATT TT-3’  P2: 5’-GTG GGA AGA TTC TCC GAA C-3’  P3: 5’-CTT TGA GAG TCA TAA ATG TGT AC-3’  P4: 5’-GGC TCC ATC TTA GTC TCC-3’ |
| *AT5G22090.2* | Forward: 5’-CTC ACT ATG CCT TGT TGC TTC-3’  Reverse: 5’-CTG ATG AAC GAC GCT ACG-3’ |

**Table S2.** Annotated introns larger than 5 kb in genes which are not protein coding in *Arabidopsis* *thaliana*.

| **Gene** | **Annotated intron** | **Length (bp)** | **Description** |
| --- | --- | --- | --- |
| *AT5G32690.1* | AT5G32690.1-1 | 57,631 | Pseudogene, homologous to *AT2G29880* |
| *AT4G10201.1* | AT4G10201.1-1 | 12,113 | Pseudogene, homologous to *AT3G21130* |
| *AT3G31005.1* | AT3G31005.1-1 | 7810 | Pseudogene, homologous to *AT1G66520* |
| *AT2G24755.2* | AT2G24755.2-5 | 5433 | Potential natural antisense gene, locus overlaps with *AT2G24750* and *AT2G24760* |
| *AT1G43781.1* | AT1G43781.1-1 | 5398 | Pseudogene, homologous to *AT1G53790* |
| *AT1G33415.1* | AT1G33415.1-6 | 5338 | Potential natural antisense gene, locus overlaps with *AT1G33420* and *AT1G33430* |
